# Heterogeneous Effects of Birth Spacing on Neonatal Mortality Risks in Bangladesh

**DOI:** 10.1111/sifp.12048

**Published:** 2018-03-06

**Authors:** Joseph Molitoris

## Abstract

The negative relationship between birth interval length and neonatal mortality risks is well documented, but heterogeneity in this relationship has been largely ignored. Using the Bangladesh Maternal Mortality and Health Care Survey 2010, this study investigates how the effect of birth interval length on neonatal mortality risks varies by maternal age at birth and maternal education. There is significant variation in the effect of interval length on neonatal mortality along these dimensions. Young mothers and those with little education, both of which make up a large share of the Bangladeshi population, can disproportionately benefit from longer intervals. Because these results were obtained from within‐family models, they are not due to unobservable heterogeneity between mothers. Targeting women with these characteristics may lead to significant improvements in neonatal mortality rates, but there are significant challenges in reaching them.

A consistent negative relationship between birth interval length and infant mortality risks has been identified in a wide variety of contexts (Cleland and Sathar 1984; Hobcraft, McDonald, and Rutstein 1985; Palloni and Millman 1986; Millman and Cooksey 1987; Pebley, Hermalin, and Knodel 1991; Boerma and Bicego 1992; Curtis, Diamond, and McDonald 1993; Rutstein 2005; Kozuki and Walker 2013; Mahande and Obure 2016; Molitoris 2017). The ubiquity of this relationship has led the World Health Organization to recommend that mothers in developing countries space their births at least three years apart (WHO 2007), and this recommendation has been implemented in community‐based family planning programs (Ahmed et al. 2015). Despite widespread agreement regarding the importance of birth spacing for improving both maternal and child health, there has been little research into the heterogeneity of this relationship. There is an emerging picture of variation in the effect of birth intervals *between* populations. It has been shown, for example, that longer birth intervals generally reduce infant mortality risks only in populations in which mortality is high (Palloni and Millman 1986; Millman and Cooksey 1987), while in low‐mortality settings there seems to be little to no consequence of birth spacing for infant health (Ball et al. 2014; Mignini et al. 2016). A recent study using historical data even showed that as the overall level of child mortality declined, the negative effect of preceding inter‐birth interval length on mortality approached zero (Molitoris 2017). This is perhaps unsurprising, since many of the theoretical mechanisms linking birth intervals to child health outcomes operate either through nutritional deficiencies or the transmission of infectious diseases (Conde‐Agudelo et al. 2012), both of which are less common in a low‐mortality context.

There has been little research, however, regarding heterogeneity in the relationship between interval length and mortality risks *within* populations. That is, do longer birth intervals disproportionately affect the chances of survival for some children over others depending on, for example, their socioeconomic circumstances or mothers’ characteristics? The identification of heterogeneous effects can assist family planning programs to more effectively target subpopulations that can derive the greatest benefit from controlling their tempo of fertility. More precise targeting may be increasingly necessary for realizing improvements in maternal and infant health as international donations to family planning programs have continued to decline in recent decades (Cleland et al. 2006), and as major donors have imposed stricter requirements on NGOs relying heavily on their foreign aid (Crane and Dusenberry 2004), exemplified by the United States government's recent decision to expand the so‐called Mexico City policy (Bingenheimer and Skuster 2017; Starrs 2017).

This article examines heterogeneity in the relationship between birth interval length and neonatal mortality risks in Bangladesh using the Bangladesh Maternal Mortality and Health Care Survey 2010 (BMMS 2010). I focus specifically on neonatal mortality because it accounts for nearly 75 percent of infant mortality in Bangladesh in 2014 and it may especially benefit the most from changes in pre‐partum behavior, like longer birth spacing. The advantage of using the BMMS over other surveys of Bangladesh, such as the Bangladesh Demographic and Health Survey, is that it has a much larger sample size (over 175,000 households), making it possible to rigorously investigate heterogeneity in population processes without losing much statistical power. Furthermore, the large sample size of the BMMS allows for estimating within‐family models, which eliminate the possibility that the results of this study are due solely to unobservable differences between mothers that could lead to biased results. In family planning circles, Bangladesh is a well‐known success story, having witnessed sustained fertility decline to near‐replacement levels and increased contraceptive uptake within a context that, until recently, had seen little economic development and continues to maintain traditional norms governing gender roles and sexual behavior. Despite enormous progress, recent DHS estimates suggest that fertility has leveled off just above replacement level and that intentions to use contraception are declining. Unlike fertility, the under‐five mortality rate has continued its remarkable fall, but it is still substantial at about 46 deaths per 1,000 population and varies widely along socioeconomic lines (National Institute of Population Research and Training (NIPORT), Mitra and Associates et al. 2016).

The present study demonstrates that the strength of the relationship between birth interval length and neonatal mortality risks varies within populations, a fact that has thus far been largely ignored and can offer valuable insight for family planning programs. First, I examine whether the relationship disproportionately affects women of certain ages. Of particular interest is how the survival prospects of children born to young women are influenced, since Bangladeshi women often begin childbearing at young ages at which they also tend to have substantially shorter birth intervals—two factors that place them at higher risk of pregnancy‐related complications. Second, I investigate differences in the relationship between birth intervals and maternal education in light of evidence suggesting that less‐educated women are at heightened risk of having shorter birth intervals and also tend to have disproportionately high rates of child mortality (De Jonge et al. 2014).

## HETEROGENEITY IN FERTILITY AND CONTRACEPTIVE USE IN BANGLADESH

Before turning to the analysis, I highlight some features of Bangladesh's demographic regime that are of central importance in discussing heterogeneity in the effect of birth spacing on mortality risks. Unless otherwise noted, the patterns and trends highlighted below come from the most recent DHS Report for Bangladesh (National Institute of Population Research and Training (NIPORT), Mitra and Associates et al. 2016). In the past four decades Bangladesh's total fertility rate (TFR) decreased from over six births per woman to 2.3, where it seems to have leveled off. The decline has occurred among women of all ages, though it was primarily women above age 40 whose fertility decreased the most with a reduction in age‐specific rates of around 90 percent. Since the beginning of the DHS program in Bangladesh in 1993, the smallest relative decrease in age‐specific fertility rates (ASFR) was among women aged 15–19. Compared to populations with similar TFRs, the ASFR for women in this age group remains very high at 113 births per 1,000 women.

It is not only the quantum of fertility that has changed, but also its tempo. The median age at first birth for women aged 20–49 has slowly increased over time, but it remains quite low at around 18.5 years. Median inter‐birth intervals have always been quite long in Bangladesh, partly due to norms promoting prolonged and nearly universal breastfeeding (UNICEF 2016); the mean duration of breastfeeding exceeds 30 months, although exclusive breastfeeding is typically terminated within six months (Giashuddin and Kabir 2004; Akter and Rahman 2010). Perhaps unsurprising, both the median age at first birth and the length of median birth intervals are positively correlated with household wealth and women's education. Even when the TFR was above six, the median interval was 33 months (Cleland et al. 1994). By 2014, this had increased to 52 months. But the latter figure obscures wide variation in birth spacing, particularly across age groups.

Nearly half of second and higher‐parity births to teenage women occur within less than two years of the preceding birth, while for older age groups only between 5 and 10 percent of intervals are so short. It is not surprising that intervals tend to be shorter in younger age groups, as there is naturally a loss of fecundity as women age; and, in the present context, women tend to start and end their reproduction at younger ages, meaning that a larger share of births to older women will be unintentional and more likely to occur after longer intervals. Contraceptive prevalence is also lower among young women for a variety of reasons. It is important to note, however, that because these statistics come from cross‐sectional surveys, the large share of short intervals among younger women is also partially a matter of selection. That is, most married women's exposure to pregnancy at ages 15–19 will generally be left‐truncated as they will tend to marry at some point in that age range. Therefore, in order to have had a second or higher‐order birth before age 20, it practically *must* have occurred within two years of the first birth. This would suggest that young women having multiple births in their teenage years may be different from those who do not. I address this issue in the empirical analysis.

The substantial decline in fertility was strongly related to the increased use of contraception, particularly modern methods (Bongaarts 2014). Contraceptive uptake has been significant in Bangladesh, rising from about 8 percent of married women aged 15–49 in 1975 to over 60 percent by 2014. The period saw not only a large increase in the adoption of contraception but also a dramatic change in the method mix used by married women. Since the first Bangladesh Fertility Survey in 1975, the share of currently using women relying on traditional methods has declined from over one‐third to 12 percent. Early in the history of family planning in Bangladesh, it was women in their 30s and 40s who adopted contraception *en masse*, and permanent sterilization was the dominant form of modern contraception promoted by family planning programs until the late 1980s (Schuler, Hashemi, and Jenkins 1995). Recent decades have seen rapid growth in the use of contraception among younger women, particularly between ages 15 and 24, and during that period oral contraceptives and injectables have become the most widely used modern methods. Nevertheless, the use of contraception in these younger age groups remains low for reasons discussed below.

The high rates of teenage childbearing in Bangladesh are largely due to a longstanding tradition of early marriage and a low rate of contraceptive use among currently married teenage women. Nearly half of all women are married before age 20, often to significantly older men; the difference in male and female mean age at first marriage was six years in 2014 (Bangladesh Bureau of Statistics 2015). Further, a large share of marriages occur before the legal age of consent, which is 18 years. Less‐educated women are also more likely to marry at younger ages.

Compared to women at older ages, married women below age 20 have a much higher unmet need for contraception. The unmet need for spacing is especially high for young women, while the unmet need for limiting family size is the lowest for this age group. Among all currently married women of reproductive age, those aged 15–19 are the least likely to be using any form of contraception, whether traditional or modern. This pattern is due to a combination of factors, which include a lower rate of cohabitation in couples with young brides, high discontinuation rates among those who practice contraception, and greater gender inequality in individual and household decision‐making for younger women.

It is not unusual for young brides to live apart from their husbands for a period of months or even years, and this may reduce exposure to pregnancy by limiting sexual contact between spouses. It may also consequently lower the demand for contraception or lead to irregular (and less effective) use. Among younger women who use contraception, oral and injectable contraceptives are the most popular choices, but discontinuation rates for both of these are among the highest of all available methods. Apart from intentions to conceive or a loss of fecundability, of which the former is common among young women, major reasons for high discontinuation rates are related to either the presence of side effects or health concerns. A qualitative study of women's postpartum perceptions of contraception in Bangladesh revealed that modern contraceptives, particularly hormonal methods, are viewed as having the potential to cause permanent damage to a woman's reproductive organs and as disruptive to the body's natural functioning (Salway and Nurani 1998). These feelings are reiterated in the latest BDHS, which shows that about 25 percent of married women below age 30 who are fecund and sexually active do not intend to use contraception because of health concerns. After the disapproval of respondents, partners, or others, health concerns are the second most common reasons for not intending to use contraception among younger women.

Married teenage women also have less influence on both individual and household decisions compared to older women. Many of these women seem to have had limited freedom even in the choice to get married in the first place. Among women marrying before reaching the legal age, it appears that nearly 60 percent would have preferred to marry later. Within marriage the story is much the same. Even concerning a woman's own health care, women below age 20 are less likely to report having the freedom to make decisions on such matters without their husbands’ input. These limitations on women's freedom have often been linked to the system of *purdah—*institutions that push women into social, economic, and physical seclusion (Amin 1997). This lack of autonomy will also limit young women's ability to adopt contraceptives from health facilities and contribute to a higher unmet need for contraception. This is clear from the fact that women who report participating in fewer major household decisions are less likely to use any contraceptive method, especially modern methods, and tend to have a greater unmet need for spacing children. Furthermore, recent work has shown that Bangladeshi women who discussed family planning with their husbands were more likely to use contraceptives (Kamal and Islam 2010). It is unclear, however, whether this is because their husbands are more supportive of their choices or are merely ambivalent, thereby retaining the power to blame wives for negative consequences of their choices (Schuler, Hashemi, and Jenkins 1995).

Women's educational attainment does not appear to be related to their involvement in family decisions, but there are clearly different expectations of a woman's role in a relationship across different levels of education, and this may have implications for their choices regarding contraception. Less‐educated women are more likely to view their husband as a dominant figure in a relationship. For instance, one‐third of women with no education believe that their husband is justified in administering corporal punishment if they displease him in various ways; among highly educated women, this figure is 16 percent. Women who are more likely to justify corporal punishment are also more likely bear the responsibility for contraception in a relationship. These women are more likely to rely on temporary modern female contraceptives, and their husbands are less likely to use male contraceptives. In addition, less‐educated women tend to rely to a greater extent on traditional methods.

## HETEROGENEOUS EFFECTS OF CAUSAL MECHANISMS

The three most commonly explored mechanisms linking birth interval length and neonatal mortality are maternal depletion, sibling competition, and the transmission of infectious disease. These have been covered in detail elsewhere (Conde‐Agudelo et al. 2012), but a brief description will highlight why interval length may be expected to affect children's outcomes to varying degrees depending on other characteristics.

The maternal depletion hypothesis argues that short intervals do not allow women to fully recover their nutritional stores after a prior birth, which may lead to diminished fetal growth as their bodies compete with that of the fetus for nourishment (Winkvist, Rasmussen, and Habicht 1992; Gibbs et al. 2012). The sibling competition hypothesis claims that closely spaced siblings will compete for similar resources from their parents, thereby diminishing their parents’ investments per child, such as the quality or quantity of calories available to each child or a certain level of cleanliness in the home environment. Competition of this nature may lead to a weaker immune system for index children, placing them at greater risk of infection and even death. The disease transmission hypothesis argues that closely spaced siblings will be more likely to pass infections to one another and, because a younger child will generally have a less‐developed immune system than its older sibling, he or she will be more likely to die at a young age. While the present analysis does not distinguish between these causal mechanisms, they should nevertheless be useful for predicting variation across subpopulations in the strength of the effect of birth intervals on neonatal mortality risks. Given these mechanisms, I briefly discuss how one could expect heterogeneity in the effect of birth intervals on mortality risks to appear along the dimensions of interest: maternal age and maternal education.

Of the three above‐mentioned causal mechanisms, we should expect maternal age to be directly linked only to the maternal depletion hypothesis. That is, there is no reason to expect a mother's age to be connected to sibling competition or infection transmission other than through socioeconomic factors. Because of their biological or gynecological immaturity, young mothers, particularly those conceiving close to menarche, tend to have several high‐risk characteristics not found in older mothers (King 2003). These include limited amino acid production in the third trimester (Thame et al. 2010), under‐developed reproductive organs, and higher risks of conditions associated with neonatal mortality, such as pre‐eclampsia and anemia (Scholl, Hediger, and Belsky 1994; Duley 2009). Under the maternal depletion mechanism, one would therefore expect longer intervals to be more beneficial to young women, particularly teenage women, since longer intervals may allow them to both recover and mature.

With regard to socioeconomic background, there is no sure way to differentiate between these three mechanisms, as they would all operate through differential exposure either to nutritional deficiencies or to disease. One would nonetheless expect that these factors would be disproportionately unfavorable for women in less advantageous socioeconomic conditions. Previous research has shown clear nutritional differences across educational groups in Bangladesh; maternal education is strongly inversely related to the risk of both childhood stunting and maternal underweight (Rahman and Chowdhury 2007; Semba et al. 2008; Campbell et al. 2010). Further, there are large differences across educational groups in children's vaccination coverage (National Institute of Population Research and Training (NIPORT), Mitra and Associates et al. 2016). Regardless of which mechanism is operating, one would expect that the marginal benefit of increasing birth intervals would be greater for less‐educated women than for highly educated women. Longer intervals would allow women of lower socioeconomic status to recover fully from the preceding birth, provide better nutrition for their children, and limit their children's exposure to higher risks of infection from siblings. There is thus ample reason to expect that the relationship between birth intervals and neonatal mortality would be disproportionately strong for women of low socioeconomic standing.

## DATA

The data used in this study come from the cross‐sectional Bangladesh Maternal Mortality and Health Care Survey 2010 (BMMS 2010), a nationally representative sample of about 175,000 households that includes 180,000 ever‐married women aged 13–49. This is a substantially larger sample size than that found, for example, in the Bangladesh Demographic and Health Surveys, the latest of which had a sample size of nearly 18,000 ever‐married women in 2014. The large sample in the BMMS 2010 was deemed necessary to accurately identify changes in maternal mortality, a relatively rare demographic event. The data included information on a wide variety of topics related to maternal health care use, demographic characteristics, socioeconomic conditions, and fertility. Although maternal mortality was the survey's primary focus, it also included basic demographic information about all of the women's children. In total, this survey provides information about some 470,000 children born to the women in the sample, and it is these children who are the main unit of analysis here. The quality of the neonatal mortality information in the BMMS 2010 is comparable to that of the BDHS; the two surveys provide very similar estimates of neonatal mortality rates for the same years.

The sample used in this study was restricted to non‐firstborn children for women who had at least three births. The omission of firstborns is due to the fact that the main independent variable of interest is the length of the preceding inter‐birth interval, which is naturally undefined for the first birth. Restricting the sample to women with at least three births was necessary as the analysis uses a within‐family design that accounts for unobserved heterogeneity between mothers. The sample was also restricted to women aged 30 or older at the time of the survey to avoid including women who were unusually highly parous at young ages. Children born following intervals of less than six months were also excluded, as it was unclear whether these intervals were real or errors in the data. In addition, children born following intervals longer than ten years were excluded, as intervals longer than this are extremely rare and are likely to skew results. Only 1.8 percent of all higher‐order births were excluded from the analysis because of an anomalous interval length. Of the excluded births, nearly all (94 percent) were births occurring after an interval of more than ten years. Children born either prior to 1975 or after 2009 were also excluded. Just a handful of children had birthdates occurring before 1975. Children born after 2009 were excluded because the collection of data occurred in 2010 and some children's exposure to neonatal mortality was truncated for that year. This led to an analysis sample size of about 240,000 children born to nearly 70,000 women. The ever‐married cohorts born in 1980 or earlier had given birth, on average, to at least three children, so the restrictions regarding family size do not restrict the sample to an unusual group of women, as would be the case if this restriction were imposed on younger cohorts who tend to have preferences for smaller families.

## METHODS

The relationship between preceding birth interval length and neonatal mortality is estimated using linear probability models with maternal fixed‐effects (FE), such that:

(1) $$Y_{ij}=S_{ij}\beta_{1,ij}+X_{ij}\beta_{k,ij}+\theta_{j}+\varepsilon_{ij}$$

The outcome variable, *Y_ij_*, is a binary variable taking the value of 1 if the child died within the first 28 days of life and 0 otherwise. The main independent variable, *S_ij_*, is a continuous variable measuring the inter‐birth interval in years between the index child's birth at parity *n* and the birth of his or her older adjacent sibling born at parity *n–*1. The birth interval variable was assigned a cubic functional form to account for the well‐documented non‐linear relationship between birth interval length and neonatal mortality risks (Hobcraft, McDonald, and Rutstein 1985; Rutstein 2005). This approach is preferable to treating the variable as a stepwise categorical variable, which would require including a large number of parameters and run the risk of overfitting the relationship between the variables of interest. **X** is a vector of individual‐specific control variables, namely the mother's age at the index child's birth, the child's birth year, birth order, multiplicity of birth (i.e. singleton or multiple birth), sex of the child, and number of siblings alive at the time of birth. Mother's age at birth and the child's birth year were included as cubic and quadratic functions, respectively, to account for the nonlinear relationship between neonatal mortality risks and maternal age (both older and younger mothers tend to have higher risks of neonatal mortality) and cohorts to account for the fact that mortality has declined in Bangladesh. Despite detailed information on health service use and access in the BMMS 2010, this study excludes these data for two reasons. First, many such questions were collected only for pregnancies occurring since October 2004 (i.e. about five years prior to the survey), which would lead to the exclusion of nearly 90 percent of the observed births from the analysis. Second, many questions in the survey concerning antenatal care referred only to the last pregnancy, and their inclusion would therefore prohibit the use of a within‐family design and consequently lead to biased estimates. The descriptive statistics for the model's covariates are given in Table 1.

**Table 1:** Descriptive statistics of analysis sample from BMMS 2010

	Mean	Standard deviation	Minimum	Maximum
Children's characteristics				
Neonatal mortality (percent dying within 28 days of birth)	4.9			
Length of preceding inter‐birth interval (in years)	3.2	1.7	0.75	10
Birth year	1994.8	7.1	1975	2009
Birth order	3.6	1.6	2	16
Sex (percent female)	49			
Multiplicity (percent twin)	1.8			
Siblings alive at birth	2.2	1.4	0	11
Maternal age at birth	25.8	5.3	13.2	48.8
Maternal characteristics				
Age at first birth	18.4	2.9	12	39.8
Birth year	1969.8	6	1960	1980
Maternal education				
No education	51.7			
Some primary	18.1			
Primary	13.1			
Some secondary or higher	17.1			
No. of children	239,272			
No. of mothers	68,039			

The models use the within‐estimator to control for unobserved heterogeneity across mothers, which may simultaneously influence both the interval length of a child and his or her probability of dying. For this reason, the error term is divided into a family‐specific component, $\theta_{j}$, and an individual‐specific component, $\varepsilon_{ij}$. This is a necessary adjustment since women who tend to have shorter intervals may also tend to have disproportionately higher risks of child mortality for unobserved reasons. For example, women who do not breastfeed at all, breastfeed for shorter durations, or breastfeed only in combination with other feeding practices may be at greater risk of conceiving a child shortly after birth, and their children may be at greater risk of dying from diarrheal diseases. Recent work has argued that taking unobservable maternal heterogeneity into account is necessary to avoid introducing omitted variable bias into estimates of the relationship between birth intervals and mortality risks of children (Zenger 1993; DaVanzo et al. 2008; Ball et al. 2014; Molitoris 2017). It is for this reason that the within‐estimator is preferred over other control strategies, such as random effects models, which assume no such correlation between individuals’ unobserved characteristics and the model covariates. The models therefore refer to variation *within* mothers, thereby eliminating the possibility that estimates are a result of compositional differences between short and long spacers. For comparative purposes, results from the same models without maternal fixed effects are also presented.

To investigate how the effects of inter‐birth intervals varied by mothers’ age at birth, the model included an interaction term between previous interval length and age at birth. It was not possible to study heterogeneity in this relationship across educational groups with interaction terms, however, as maternal education does not vary across children and therefore cannot be estimated using the within‐family design. Instead, the models were stratified by mothers’ educational attainment. Four separate models were estimated to account for each education category in the survey: no education, some primary, completed primary, and some/completed secondary or higher. Based on the above‐mentioned models, average marginal effects of birth interval length were then estimated across different values of the interacted variable. This allows one to investigate the effect of “adding” an additional year to an average birth interval on the risk of neonatal mortality at different ages at birth and across various levels of education. In the absence of heterogeneity in this effect, one would expect that marginal effects of birth interval length should be equal regardless of maternal age or education. If some groups benefit more greatly from longer intervals, one would expect a larger negative effect of increasing birth interval length on the probability of a child dying for some groups than for others.

## RESULTS

### Average Effects of Preceding Interval Length on Neonatal Mortality Risks

In order to provide a comparison to previous research, the basic model was first estimated without interaction terms and with a categorical operationalization of preceding interval length (see online Appendix).^1^ As has been found previously, there is a negative, non‐linear relationship between interval length and the risk of neonatal mortality. In the OLS model, the risk of mortality declines as birth interval length increases, and there is no reversal in this trend. Once taking unobserved heterogeneity into account in the FE models, however, the story changes somewhat. First, the average effect of interval length on neonatal mortality risks is considerably smaller than the OLS estimates would indicate. This is true for any given interval length. Nevertheless, there is still a statistically significant effect and it is also substantively meaningful. Second, although longer intervals are associated with lower neonatal mortality, the effect sizes are diminished by nearly half, suggesting that important unobserved factors partially account for this relationship. Third, once birth intervals exceed 47 months, the mortality‐reducing effect begins to turn upward. This result, shown in previous research, led to the WHO recommendation of spacing children about three years apart (Rutstein 2005). Having established that Bangladesh's population, on average, fits to the expected patterns of birth spacing on child mortality, I now investigate heterogeneity in this relationship.

### Variation across Maternal Age

Table 2 presents the estimated marginal effect of an increase in birth interval length from two years to three years for a woman of median childbearing age (22 years) on the probability of neonatal mortality. This is the average effect estimated from the interaction model of interval length and maternal age at birth. The marginal effects were evaluated at an interval length of two years for two reasons. First, among short intervals this is the most common interval length. Second, these are the individuals who would be directly influenced by following the WHO recommendation of having an interval between three and five years long. The marginal effects were also converted to the equivalent percent reduction in neonatal mortality to facilitate interpretation. When stratified by education groups, the percent reduction was calculated based on the group‐specific probabilities of neonatal mortality for children born following a two‐year interval. The table also presents the results of a test for joint significance of the interaction terms and a likelihood ratio (LR) test to identify whether the interaction model provides a better fit to the data compared to the model with no interaction. The results for the pooled model (i.e. all education groups) show that an increase in interval length from two years to three for a woman giving birth at the median age at birth decreases her child's probability of neonatal death by 0.015, which translates to a 27.6 percent decrease in mortality for children born following intervals of around two years. The F‐test and LR test both confirm that there is a significant interaction between age at birth and length of the birth interval.

**Table 2:** Average marginal effect of an increase in interval length from two years to three years, tests of joint significance of interaction terms, and goodness‐of‐fit comparison for models with and without interaction

	Average marginal effect	Standard error	Relative reduction in probability of dying (percent)	Wald F‐test	Likelihood ratio test
Interval × age at birth (all groups)^a^	–0.015***	0.001	–27.6	25.4***	75.6***
No education	–0.017***	0.002	–28.4	17.8***	55.5***
Some primary	–0.015***	0.003	–28.8	5.1***	12.6
Primary	–0.020***	0.004	–44.9	6.1***	27.8***
Some secondary or higher	–0.004	0.003	–10.3	3.0***	48.4***

NOTE: ^*^p<0.1 ^**^p<0.05 ^***^p<0.01. Average marginal effects (AME) were evaluated at an interval length of two years. Thus, AME refers to the effect of increasing an interval from two years to three years on the probability of neonatal death. The Wald F‐test is a test of the joint significance of the interaction terms included in respective models. Statistical significance indicates that the estimates of the interaction term are different from zero. The likelihood ratio (LR) test is a test comparing the goodness‐of‐fit between models with and without the interaction. Statistical significance indicates a better goodness‐of‐fit for the interaction model. The joint significance tests and goodness‐of‐fit tests for the two interactions are presented as pooled models and also stratified by education. When the models are pooled with all education groups, the LR test is a comparison between models with and without the specified interaction for all individuals. When the models are stratified by education group, the LR test is a comparison between models with and without an interaction term only for the respective education group.

a Evaluated at age at birth of 22 years, the median age at birth in the sample.

**Figure 1:**
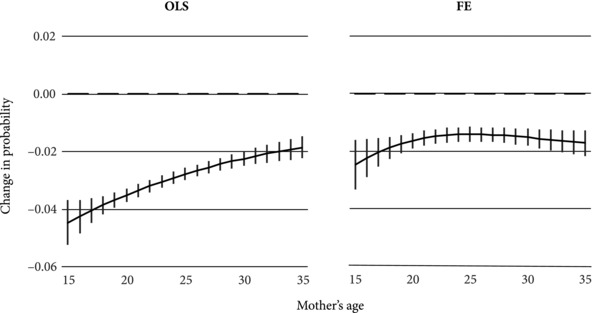
Marginal effect of birth interval length on risk of neonatal mortality, by maternal age at birth NOTE: Figures reflect the change in the probability of neonatal mortality associated with a one‐year increase in the length of the preceding inter‐birth interval. The left panel presents predictions under the assumption that there is no correlation between family‐specific unobserved heterogeneity and the length of the preceding interval. The right panel presents predictions after adjusting for maternal fixed effects. All other covariates held at their mean values.

To better understand the nature of the interaction between age at birth and interval length, the average marginal effects of increasing an interval were then plotted across maternal ages. The marginal effects of interval length on mortality risks were calculated only for mothers giving birth between ages 15 and 35 as a low number of births occurred at older ages. It is clear that longer birth intervals benefit mothers of certain ages over others (Figure 1). In both the OLS and FE models, there was a nonlinear relationship between interval length and neonatal mortality by maternal age. Increasing the length of the preceding birth interval for children born to young mothers, particularly those below age 25, would have a greater mortality‐reducing effect than at older ages. This is a significant finding, as these are ages at which the majority of Bangladeshi women have already progressed to higher‐order births. In both the OLS and FE models, the marginal effect of increasing birth intervals was large for teenage women. The FE predictions suggest that a one‐year increase in interval length could reduce neonatal mortality risks by about 0.015, compared to about 0.025 in the OLS models. Above the mid‐20s, the effect of increasing interval length was more or less unchanged, but remained negative, implying that there is also a benefit for children whose mothers gave birth at later ages. To put these figures in perspective, the marginal effect estimated from the FE models is equivalent to about a 20 percent reduction in the probability of dying in the first 28 days of life.

**Figure 2:**
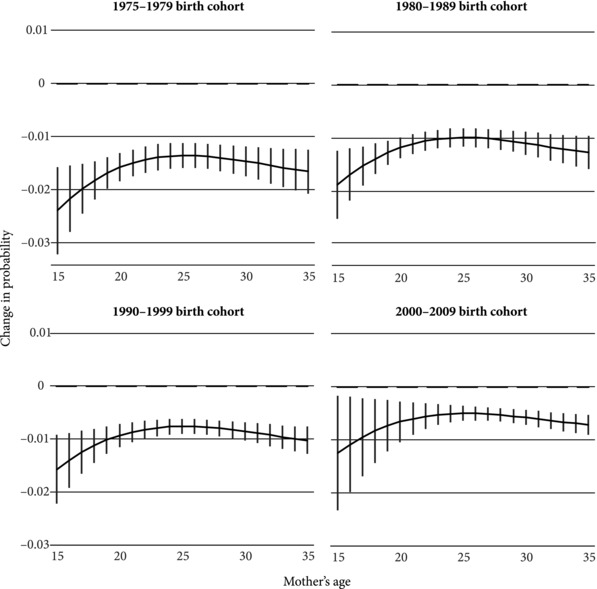
Marginal effect of birth interval length on risk of neonatal mortality, by maternal age at birth and child's birth cohort NOTE: Figures reflect the change in the probability of neonatal mortality associated with a one‐year increase in the length of the preceding inter‐birth interval. The panels refer to births occurring within the specified years. All covariates held constant at their mean values. Estimates based on within‐family models.

Figure 2 stratified this analysis to examine how the relationship between interval length and age has changed over time. These figures refer only to the FE estimates. The nonlinear nature of this relationship has clearly persisted across birth cohorts, though it appears to be weakening. This may be a result of general improvements in nutrition or in the epidemiological environment, which may reduce the strength of the infection and maternal depletion mechanisms. Although the magnitude of the effect has been decreasing for births to women of all ages and for all cohorts, longer intervals are clearly associated with larger reductions in neonatal mortality for younger mothers than for older mothers. For children born in the 1970s, increasing a birth interval by one year for a teenage mother would have reduced neonatal mortality risks by over 0.02. The effect on a child born to a young mother between 2000 and 2009 was only about 0.012. The curves can be seen slowly moving toward zero over time, especially for women giving birth in their mid‐20s. Nevertheless, the disproportionate benefit for teenage mothers persists.

### Variation across Maternal Education

Returning to Table 2, we can see the average effect of increasing a birth interval from two years to three years for different educational groups. The most noteworthy finding here is that women who have advanced beyond primary education seem to experience no benefit from increasing the length of their birth intervals. Women with less than secondary education, however, could reduce their children's neonatal mortality risks significantly by increasing spacing. For women with less than a primary degree, an increase in interval length from two years to three was associated with a reduction in neonatal mortality risks of about 28 percent. For those with a primary degree, the same increase in spacing would reduce neonatal mortality by almost 45 percent. It is not clear why the absolute and relative influence of interval length is greatest for women having only a primary degree and not for less‐educated women. Nevertheless, there is a clear distinction between women who have studied beyond primary school and those who have not. Substantial reductions in mortality can be gained from increasing birth intervals for the former group, while spacing may be of little consequence for the latter.

**Figure 3:**
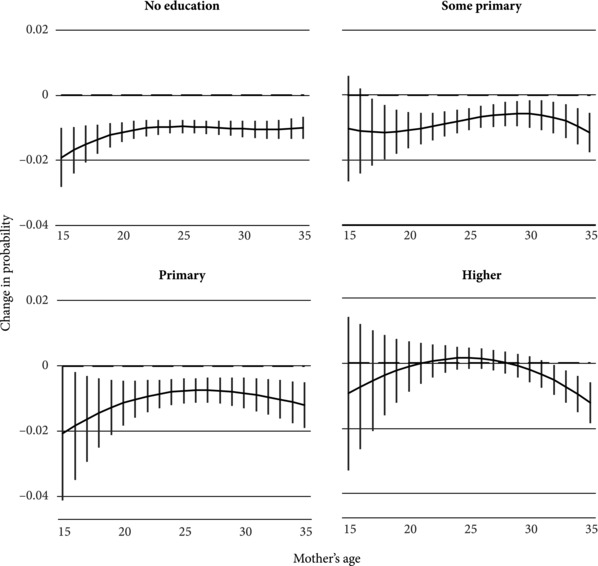
Marginal effect of birth interval length on risk of neonatal mortality, by maternal education and age at birth NOTE: Figures reflect the change in the probability of neonatal mortality associated with a one‐year increase in the length of the preceding inter‐birth interval. The panels refer to births occurring within the specified years. All covariates held constant at their mean values. Estimates based on within‐family models stratified by maternal education.

Figure 3 again shows the effects of interval length by maternal age, now stratified by maternal education. The findings in this graph reiterate that there is a clear difference in the effect of birth intervals on neonatal mortality for women with more than primary education compared to those with a primary degree or less. In general, we see the same pattern that was shown in Figure1: birth intervals reduce mortality the most for teenage mothers, though there seem to be mortality‐reducing effects of birth intervals at older ages as well. The difference in this figure, however, is that these effects are more or less confined to women with a primary degree or less. For women with more than primary education, a significant effect can be identified only for those giving birth in their 30s, when few births occur. Otherwise, the estimates are statistically indistinguishable from a null effect.

## DISCUSSION

The strength of the mortality‐reducing effect of birth intervals varies along two important dimensions: maternal age and maternal education. These variables were of special interest because the risk of experiencing short intervals is not equally distributed across ages and educational backgrounds and because they are relatively straightforward to incorporate into the targeting strategies of a family planning program.

An important source of heterogeneity observed in this study is that this relationship is highly dependent on a mother's age at birth. A benefit of longer intervals on mortality risks was found among all age groups, indicating that it is a necessary element in any family planning initiative. Yet a consistent finding was that young mothers could benefit the most. The children of teenage mothers and mothers in their early 20s would experience the largest gains in survival probabilities from longer birth intervals. The age‐specific pattern, despite changes in the magnitude of the relationship, was consistent across birth cohorts. This is no trivial result, as Bangladeshi women give birth at very young ages. According to the 2014 Bangladesh DHS, almost 40 percent of currently married women under 25 had already progressed to their second or higher‐order birth (National Institute of Population Research and Training (NIPORT), Mitra and Associates et al. 2016). This risk factor is thus highly relevant to the Bangladeshi context.

Another significant finding was that the survival prospects of children born to less‐educated women are influenced to a greater degree by the length of the preceding birth interval. Whether this is due to greater exposure to disease, poorer maternal nutrition, or a combination of the two is unclear, however. Nevertheless, these effects were again the strongest for women giving birth at young ages. At older ages, there did not appear to be any substantial differences across education groups other than for the most highly educated. It is important to realize that a substantial share of Bangladesh's female population falls into this low education group. Among Bangladeshi children attending school today, about 50 percent still do not progress beyond primary school (Bangladesh Bureau of Statistics et al. 2017). This means that, despite remarkable gains in educational expansion in the country, a large share of future parents will fall into groups for whom interval length will be an important consideration for their children's well‐being.

These findings have implications for family planning strategies in Bangladesh. First and foremost, the findings reinforce the necessity to communicate the importance of birth spacing to all women, as there was a significant mortality‐reducing effect of longer intervals. This recommendation has gained momentum in recent years and should continue to be a part of communication strategies. There is now substantial research in support of this position (see Conde‐Agudelo et al. 2012). Until now, however, it had not been shown that some individuals stand to gain even more from birth spacing than others.

The second major implication is that this information must be more intensely targeted at young women and their husbands. Women giving birth below age 25 saw larger reductions in neonatal mortality rates associated with birth interval length than those at older ages. There are some unique challenges to reaching these women, however. They tend to have the greatest unmet need for family planning for birth spacing, yet the traditions of teenage marriage and patriarchy in Bangladesh restrict young women's decision‐making capabilities. Teenage married women are much less likely to be able to visit health facilities without being accompanied by their husband, for example. Family planning programs in Bangladesh have indeed acknowledged these issues for many decades. Learning from early failed attempts to increase contraceptive uptake in the 1970s (Cleland et al. 1994), family planning programs in Bangladesh may be viewed as an example of a successful attempt to fit programs to local cultural norms in order to maximize their effectiveness. They have attempted to address these norms through the household delivery of contraceptive advice and services by literate female workers and by galvanizing the support of local elites and religious leaders (Simmons et al. 1988; Cleland et al. 1994).

Despite achieving much progress, challenges remain in addressing the power imbalances within relationships that often lead to higher unmet among young women and women with less education. Generally speaking, two approaches are adopted to address power imbalances: woman‐centered and couple‐centered approaches. Most family planning strategies have been largely focused on women and attempt to encourage family planning by circumventing monetary and social costs of contraception through service delivery, mass media campaigns, social marketing, and the increased supply of contraceptives. These initiatives should not be overlooked, because their impact has been substantial (Bongaarts 2014), but this approach has been criticized given that many goals of modern family planning programs, like those concerning maternal and child health, require the active participation of both spouses (Becker 1996). Furthermore, the woman‐centered approach emphasizes using contraceptives that require only women's participation, such as hormonal methods, which are often viewed with skepticism and caution as they are perceived as disrupting bodily processes (Salway and Nurani 1998). A woman‐centered approach is also weaker in addressing certain barriers to the practice of family planning. Because of married women's overwhelming dependence on their husbands in Bangladesh, the threat of divorce or desertion weighs heavily on any decision that may violate their husband's wishes. Even the perception that a husband is opposed to family planning has been shown to dissuade a young woman from adopting contraception, and the apparent apathy of men toward their wives’ contraceptive choices does little to quell these concerns (Schuler, Hashemi, and Jenkins 1995).

One possibility for addressing gender inequality in relationships is to reduce women's financial dependence on men. A common tool thought to achieve this is microcredit, which has been issued extensively in Bangladesh. One well‐known study has shown that access to microcredit empowered Bangladeshi women by increasing their economic contribution to household, political, and legal awareness, physical mobility, and political participation (Hashemi, Schuler, and Riley 1996). But evidence supporting the empowering effect of microcredit is mixed. It has been shown, for example, that microcredit transfers tend to be appropriated by husbands and ultimately do little to increase women's independence (Goetz and Gupta 1996). This approach may be ineffective without addressing the cultural roots of the patriarchal system that restricts young women's ability to satisfy their demand for contraception.

An alternative possibility is to treat men's behavior and attitudes as equal in importance to those of women in family planning programs, and this has resulted in attempts to enlist men's support by including them in programs’ overarching strategies. Previous research has shown that Bangladeshi women who have their husbands’ approval for family planning or are in more equalitarian relationships are more likely to adopt contraception and less likely to have an unmet need for contraception (Kamal 2000; Kamal and Islam 2010; Uddin, Pulok, and Sabah 2016). It has been a challenge, however, to engage husbands, which may be especially important when discussing young married women who have significantly less bargaining power in a relationship compared to those at older ages. The difficulty of fostering men's participation is not unique to Bangladesh, and has been observed in other developing contexts (Ditekemena et al. 2012). Despite some evidence suggesting that men wish to be included in these programs and are eager to learn more about family planning (Blanc 2001), it is unclear whether this approach is actually a more effective strategy for promoting contraceptive use than targeting women alone. In certain contexts, men who are included in family planning strategies tend to more openly communicate with their wives and have greater levels of contraceptive use (Wang et al. 1998; Shattuck et al. 2011), but in other contexts there seems to be no advantage over women‐centered strategies (El‐Khoury et al. 2016). There have been relatively few randomized control trials of these sorts of interventions in modern developing contexts, however. This may be a promising approach for addressing the cultural roots of gender inequality within marriages that lead to greater unmet need among young and less‐educated women. In turn, this approach could have a substantial impact on neonatal mortality rates by increasing mean birth intervals among younger women.

## CONCLUSION

This study is the first to show that the well‐known relationship between birth spacing and neonatal mortality is not uniform across ages or educational groups. Considerable heterogeneity in this relationship exists, and there are specific groups to whom the importance of birth spacing must be communicated. Children born to young and less‐educated women are disproportionately negatively affected by the length of the preceding birth interval. By using a within‐family approach, the possibility that these differences are caused by unobserved compositional differences across mothers is eliminated. In other words, the differences in the magnitude of the effect across ages and education are not driven by the fact that mothers giving birth at young ages or with low education somehow differ from mothers with different characteristics for unobserved reasons. The results suggest that family planning programs that more specifically target these high‐risk groups may help to accelerate the decline of neonatal mortality. There are serious challenges in reaching these groups, however, as strong norms of patriarchy tend to disproportionately limit the freedom of these women. Future family planning strategies will need to find a way to address this issue.

## Supporting information

Appendix

## Footnotes

1 Appendix is available at the supporting information tab at wileyonlinelibrary.com/journal/sfp.
