# Supplementary material for: Heterogeneous Effects of Birth Spacing on Neonatal Mortality Risks in Bangladesh
Source: Stud Fam Plann. 2018 Mar 6;49(1):3–21. doi: 10.1111/sifp.12048 (PMC5947260; doi:10.1111/sifp.12048)
Supplement: Supplementary file 1 — Appendix [file SIFP-49-3-s001.docx]

## APPENDIX

Estimates from OLS and Mother FE models of birth interval length on neonatal mortality risk.

|  | OLS | Mother FE |
| --- | --- | --- |
| *Preceding Interval Length* |  |  |
| <18 months | (ref) | (ref) |
| 18-23 " " | -0.027*** | -0.015*** |
| 24-29 " " | -0.038*** | -0.019*** |
| 30-35 " " | -0.046*** | -0.022*** |
| 36-41 " " | -0.056*** | -0.027*** |
| 42-47 " " | -0.057*** | -0.029*** |
| 48-53 " " | -0.059*** | -0.028*** |
| 54-59 " " | -0.057*** | -0.023*** |
| 60+ " " | -0.057*** | -0.021*** |
| Age at Birth | -0.025*** | -0.021*** |
| Age at Birth^2^ | 0.001*** | 0.001*** |
| Age at Birth^3^ | 0.000*** | 0.000*** |
|  |  |  |
| Birth Year | -0.001*** | 0.004** |
| Birth Year^2^ | 0.000*** | 0.000*** |
|  |  |  |
| *Birth Order* |  |  |
| 2 | (ref) | (ref) |
| 3 | 0.013*** | -0.157*** |
| 4 | 0.033*** | -0.308*** |
| 5 | 0.052*** | -0.453*** |
| 6 | 0.070*** | -0.593*** |
| 7 | 0.088*** | -0.727*** |
| 8 | 0.103*** | -0.860*** |
| 9 | 0.122*** | -0.980*** |
| 10+ | 0.137*** | -1.100*** |
| *Sex* |  |  |
| Male | (ref) | (ref) |
| Female | -0.012*** | -0.010 |
| *Multiplicity* |  |  |
| Twin | (ref) | (ref) |
| Singleton | -0.259*** | -0.124*** |
| Siblings Alive at Birth | -0.025*** | 0.165*** |
|  |  |  |
| Constant | 0.627*** |  |
| Mothers | 79,605 | 79,605 |
| Children | 267,605 | 267,605 |
| F-statistic | 395.1 | 878.3 |
| R^2^ | 0.043 | 0.123 |

Note: * p<0.1 ** p<0.05 *** p<0.01. R^2^ for mother FE model refers to within-R^2^. Models estimated as Linear Probability Models. Models used in analysis use a continuous operationalization of the preceding interval length. The variable was categorized here to aid interpretation.
